# Effects of Different Dietary Levels of Chia ( *Salvia hispanica* L.) and Quinoa ( *Chenopodium quinoa* Willd.) on Adipokines and Anthropometric Parameters in Rats

**DOI:** 10.1002/fsn3.72325

**Published:** 2026-09-05

**Authors:** Kübra İspir, Sevinç Yücecan, Ilyas Bozkurt, Halit Imik

**Affiliations:** ^1^ Department of Nutrition and Dietetics, Institute of Health Sciences Lokman Hekim University Ankara Turkey; ^2^ Department of Nutrition and Dietetics, Faculty of Health Sciences Bingol University Bingol Turkey; ^3^ Department of Nutrition and Dietetics, Faculty of Health Sciences Lokman Hekim University Ankara Turkey; ^4^ Department of Pharmacy Services, Nihat Delibalta Göle Vocational High School Ardahan University Ardahan Turkey; ^5^ Department of Animal Nutrition and Nutritional Diseases, Faculty of Veterinary Medicine Atatürk University Erzurum Turkey

**Keywords:** adipokine, anthropometric measurements, body mass index (BMI), chia, quinoa

## Abstract

Chia ( *Salvia hispanica* L.) and quinoa ( *Chenopodium quinoa* Willd.) are pseudocereals that stand out due to their high nutrient density and functional component content. Their richness in protein, dietary fiber, unsaturated fatty acids, vitamins, minerals, and bioactive phytochemicals makes them important dietary components with potential roles in metabolic processes and inflammation‐related mechanisms. This study aimed to evaluate the effects of chia and quinoa seeds, included at 5%, 10%, and 20% in isocaloric and isonitrogenous diets, on serum adipokines (omentin, visfatin, resistin, chemerin), insulin, anthropometric measurements, and body mass index (BMI) in healthy, normal weight male Wistar rats, relative to a control diet. Seventy male Wistar albino rats were divided into seven groups: control diet, 5%, 10%, and 20% chia seed diets (LD‐CD, MD‐CD, HD‐CD) and 5%, 10%, and 20% quinoa seed diets (LD‐QD, MD‐QD, HD‐QD). After 6 weeks, serum adipokine and insulin levels were measured. Weekly body weight, body length, abdominal circumference, and chest circumference were recorded, and BMI values were calculated. Chia and quinoa supplementation altered serum adipokine and insulin levels, with the observed responses varying according to the supplementation level. High‐dose (20%) supplementation decreased serum chemerin, resistin, and insulin levels while increasing omentin levels compared with controls. Moderate (10%) and high (20%) supplementation altered serum visfatin levels. Chia and quinoa supplemented diets reduced BMI compared with controls. The HD‐CD group also showed lower body weight gain and abdominal circumference. Including chia and quinoa seeds as dietary alternatives may positively affect serum adipokine levels and anthropometric parameters.

## Graphical abstract

Six weeks dietary supplementation with chia ( *Salvia hispanica* L.) and quinoa ( *Chenopodium quinoa* Willd.) modulated adipokine profiles and anthropometric parameters in male Wistar rats: 20% chia and quinoa decreased serum chemerin, resistin, and insulin levels while increasing omentin; 10%–20% supplementation decreased visfatin; all supplemented diets reduced BMI and 20% chia attenuated body weight gain and increases in abdominal and chest circumferences.

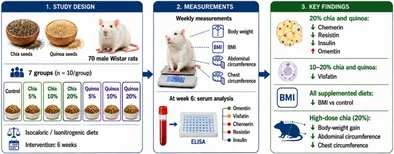

**Abbreviations**
20‐HE: 20‐hydroxyecdysone
ANOVA: analysis of variance
BMI: body mass index
ELISA: enzyme‐linked immunosorbent assay
HD‐CD: high‐dose chia diet
HD‐QD: high‐dose quinoa diet
LD‐CD: low‐dose chia diet
LD‐QD: low‐dose quinoa diet
MD‐CD: moderate‐dose chia diet
MD‐QD: moderate‐dose quinoa diet
NFE: nitrogen‐free extract
SD: standard deviation
SEM: standard error of the mean
SPSS: statistical package for the social sciences

## 1 Introduction

Chronic diseases are known as noncommunicable diseases that pose significant challenges for society and healthcare services (Ma et al. 2025). These noncommunicable diseases are closely associated with high mortality rates (Jangra et al. 2025). The increasing aging population worldwide, urbanization, lifestyle changes, and especially the increase in the production and consumption of high‐carbohydrate cereals in recent years are leading to an increase in metabolic disorders in societies and consequently to the emergence of chronic diseases, primarily obesity (Ma et al. 2025; Nandan et al. 2024). While cereals are an integral part of many diets, deficiencies in vitamins, minerals, and many bioactive nutrients are associated with negative health outcomes (Bekkering and Tian 2019).

In recent years, adopting healthy lifestyles has become increasingly popular in the fight against many chronic diseases, including obesity and related illnesses (Omran et al. 2023). Increased health awareness in societies and approaches aimed at improving quality of life throughout human life have also increased awareness of the need to improve and enhance individuals' nutritional habits (Vajdovich et al. 2025). In this context, chia and quinoa seeds, possessing novel functional and bioactive properties, stand out as superfoods (Agarwal et al. 2023; Goyat et al. 2018). Chia and quinoa are two nutritious seeds rich in dietary fiber, proteins, fatty acids (especially omega‐3 fatty acids), vitamins, minerals, and polyphenols (Eshraa et al. 2024). These seeds are rich in both nutrients and bioactive components, making them protective and effective functional pseudocereals against many chronic diseases such as obesity, hypertension, cardiovascular diseases, and diabetes (Goyat et al. 2018; Lopez‐Moreno et al. 2023; Stabnikova and Paredes‐López 2024; Zhang and Li 2024).

Adipokines, secreted by adipose tissue, are biologically active molecules that exert a variety of effects on health and disease. Cytokines are responsible for regulating many physiological processes, including inflammation, metabolism, immunity, cardiovascular function, and energy homeostasis (Donato Jr., 2023). While some adipokines exhibit pro‐inflammatory properties (resistin, visfatin), others exhibit anti‐inflammatory effects (omentin) (Ambroszkiewicz et al. 2022). These cytokines regulate inflammatory responses by regulating the release of immune cells and play a role in modulating diseases or metabolic disorders that arise following the inflammatory response. In addition, adipokines can modulate the cognitive system and eating behaviors through neurotransmitters (Clemente‐Suarez et al. 2023). It has been suggested that many nutrients and bioactive compounds may have a significant impact on the production and function of adipokines (Al‐Muzafar and Amin 2017; Derdemezis et al. 2011; Sepidarkish et al. 2022).

There are no known studies examining the direct and comparative effects of quinoa and chia seeds on the adipokines omentin, resistin, visfatin, and chemerin, which are important indicators of chronic disease and metabolic health; existing studies generally consist of obese/overweight, metabolically unhealthy experimental animals and humans. To date, varying amounts of chia and quinoa supplementation have not been investigated in healthy rats with normal body weight. In our experimental model, Wistar rats were used to study metabolic regulation and adipokine responses due to their physiological similarities to humans in terms of energy metabolism and endocrine function. The aim of our study was to determine the effects of chia and quinoa supplementation in different proportions on adipokines, which are important indicators of chronic diseases and metabolic health, and on anthropometric measurements and body mass index (BMI) in healthy rats with normal body weight.

## 2 Materials and Methods

### 2.1 Animal Model

Our animal care and experimental protocols have been approved by the Atatürk University Experimental Animal Ethics Committee (Approval date: February 25, 2025#45; Document No.: E‐36643897‐000‐2500066231), which operates in accordance with European (EU) Directive 2010/63/EU. In this study, 70 male Wistar albino rats (10 weeks old) between 200 and 270 g obtained from the Laboratory Animal Facility of our university's Medical Experimentation, Application and Research Center were used. The sample size was determined by considering the 3R rule and performing G*Power analysis. All rats were of the same strain (Wistar albino) and were housed in standard plastic cages under optimal laboratory conditions (22°C± 1°C, 12‐h light/dark cycle). The rats were divided into seven groups, with 10 rats in each group based on their body weight. For the 10 days prior to the start of the experiment and throughout the experiment, the rats were provided with unlimited amounts of food and fresh water. Within each group, the 10 rats were housed in two standard cages (5 rats per cage) to maintain social housing in accordance with animal welfare requirements (EU Directive 2010/63/EU).

### 2.2 Composition and Preparation of the Diet

For this study, experimental diets were prepared consisting of seven diet groups that were isocaloric and isonitrogenous:

1. Control diet.
2. Low‐dose chia diet (LD‐CD): Diet containing 5% chia seeds.
3. Medium‐dose chia diet (MD‐CD): Diet containing 10% chia seeds.
4. High‐dose chia diet (HD‐CD): Diet containing 20% chia seeds.
5. Low‐dose quinoa diet (LD‐QD): Diet containing 5% quinoa seeds.
6. Medium‐dose quinoa (MD‐QD): Diet containing 10% quinoa seeds.
7. High‐dose quinoa diet (HD‐QD): Diet containing 20% quinoa seeds.

Commercial food grade chia ( *Salvia hispanica* L.) and white quinoa ( *Chenopodium quinoa* Willd.) seeds (Yayla Gurme, Yayla Agro Gıda Sanayi ve Ticaret A.Ş., Mersin, Türkiye; Food Business Registration No. TR‐33‐K‐001608) were purchased from a local hypermarket in Erzurum, Türkiye. The chia seeds complied with the Turkish Food Codex (Food Enterprise Registration No. TR‐33‐K‐001608), whereas the quinoa product consisted of 100% white quinoa of Peruvian origin. The batch/lot numbers of the chia and quinoa products were Lot No. 26P2253 and Lot No. 26P1920, respectively. Both products were obtained from a single commercial batch and used throughout the study to ensure consistency in the preparation of the experimental diets. The diet formulations were prepared at the Bayramoğlu Feed Mill. Table 1 shows the ingredient, nutrient, and energy composition of the experimental diets for each group, together with the dietary inclusion levels of chia and quinoa seeds.

**TABLE 1:** Ingredient, nutrient and energy composition of the experimental diets.

Feed raw materials, %	Groups						
	Control	LD‐QD	MD‐QD	HD‐QD	LD‐CD	MD‐CD	HD‐CD
Wheat	35.15	30.57	25.54	14.0	30.41	25.49	15.7
Quinoa	—	4.93	9.81	19.64	—	—	—
Chia	—	—	—	—	4.98	10.0	19.9
Wheat bran	3.20	1.87	2.51	5.0	2.67	5.84	5.8
Sunflower seed meal	27.80	27.61	27.51	27.5	27.24	27.18	34.75
Soybean meal	16.80	17.75	17.67	17.9	17.81	14.66	7.01
Corn	7.0	6.90	6.87	3.67	5.40	4.49	4.80
Soybean oil	2.42	2.20	3.69	3.10	2.28	1.71	0.10
Molasses	2.43	2.46	2.45	2.38	2.10	2.43	2.17
Marble powder	0.81	0.81	0.81	0.81	0.81	0.81	0.81
Salt	0.81	0.81	0.81	0.81	0.81	0.81	0.81
Vitamin‐Mineral Premix^a^	0.1	0.1	0.1	0.1	0.1	0.1	0.1
Dicalcium phosphate (DCP)	2.2	2.2	2.2	2.2	2.2	2.2	2.2
Corn waste	1.28	1.79	0.0	2.86	3.19	4.28	5.85
Nutrient and energy composition of the experimental diets							
Dry matter (%)	89.78	89.98	90.43	90.88	90.07	90.23	90.74
Moisture (%)	10.22	10.02	9.57	9.12	9.93	9.77	9.26
Total protein (g/100 g)	23.94	24.12	24.13	24.13	24.48	24.00	24.22
Crude fat (%)	3.67	3.64	5.38	5.23	4.89	5.84	7.10
Crude fiber (%)	6.30	6.38	6.59	7.07	7.31	8.49	11.13
Crude ash (%)	4.22	4.21	4.25	4.38	4.30	4.48	4.81
Nitrogen‐free extract (NFE) (%)	51.65	51.63	50.08	50.07	49.09	47.42	43.48
Total energy (kcal/100 g)	280.13	280.12	279.85	280.27	280.27	279.92	280.20

Abbreviations: HD‐CD, high‐dose chia diet; HD‐QD, high‐dose quinoa diet; LD‐CD, low‐dose chia diet; LD‐QD, low‐dose quinoa diet; MD‐CD, moderate‐dose chia diet; MD‐QD, moderate‐dose quinoa diet.

^a^ Contents per kilogram of premix: vitamin A, 3,600,000 IU; vitamin D_3_, 800,000 IU; vitamin E, 7200 IU; vitamin K_3_, 800 mg; vitamin B_1_, 720 mg; riboflavin, 2640 mg; calcium pantothenate, 6120 mg; niacin, 12,000 mg; folic acid, 400 mg; vitamin B_12_, 6 mg; biotin, 40 mg; choline, 100,000 mg; Mn, 39,680 mg; Fe, 20,000 mg; Zn, 33,880 mg; Cu, 4000 mg; I, 400 mg; Se, 80 mg.

The nutrient and energy contents of the experimental diets (Table 1) were determined as follows. The crude protein content of the feed ingredients was determined by the Kjeldahl method (Sáez‐Plaza et al. 2013) based on the quantification of the total organic nitrogen of each sample and its conversion into crude protein. The dry matter, moisture, crude fat, crude fiber, and crude ash contents of the experimental feeds were analyzed at Bayramoğlu Feed Mill and calculated using the nitrogen‐free extract (NFE) difference method. The energy content of the diets was not measured directly but was calculated from the proximate composition of each formulation using the Brill Multi formulation (National Research Council 1994) in accordance with the Turkish Standards Institute (TSE‐9610) and National Research Council (NRC) recommendations.

### 2.3 Experimental Design

After a 10‐day adaptation period, the rats were randomly allocated into seven groups based on body weight. The dietary inclusion levels of 5%, 10%, and 20% chia and quinoa were selected based on the supplementation ranges previously used in experimental studies (Al‐Sayed et al. 2019; Ali Saif Alyamani 2020; El‐Kholie et al. 2023; Montes Chani et al. 2018) and were designated as low, moderate, and high inclusion levels, respectively. These levels were chosen to represent low, moderate, and high dietary inclusion levels and to enable comparison of biological responses across different supplementation levels. The rats were provided with the respective experimental diets and drinking water ad libitum for 42 days. At the end of the experiment, following a 12‐h fast, all rats were anesthetized with an intraperitoneal injection of ketamine hydrochloride (80 mg/kg; Ketasol, Austria) and xylazine (10 mg/kg; Xylazinbio, Bioveta). Next, blood obtained via a fatal cardiac puncture was centrifuged at 4000 rpm for 10 min at 4°C to obtain serum, which was then stored at −80°C. No animals, experimental units, or data points were excluded from the analyses. The experimental design of the study is summarized in Table 2.

**TABLE 2:** Experimental design.

Group number	Group (*n* = 10)	Amount (%)
I	Control diet (control group)	**—**
II	Low‐dose chia diet (LD‐CD)	5
III	Moderate‐dose chia Diet (MD‐CD)	10
IV	High‐dose chia diet (HD‐CD)	20
V	Low‐dose quinoa diet (LD‐QD)	5
VI	Moderate‐dose quinoa diet (MD‐QD)	10
VII	High‐dose quinoa diet (HD‐QD)	20

### 2.4 Biochemical Analyses

#### 2.4.1 Assessment of Serum Adipokines and Insulin Levels

All serum samples were analyzed in duplicate using commercially available rat ELISA kits (FineTest, Wuhan Fine Biotech Co. Ltd., Wuhan, China) according to the manufacturer's instructions. Calibration curves were generated using the standards supplied with each kit and sample concentrations were calculated from the corresponding standard curves. Serum omentin levels were measured using a rat‐specific omentin ELISA kit (Cat. No.: FY‐ER3526, Lot No. KDF7VG51203520), with a detection range of 0.32–20 ng/mL. Serum chemerin concentrations were determined using a rat chemerin ELISA kit (Cat. No.: FY‐ER2076, Lot No. KDK1AV51203519) and the reported detection range of the method was 31.25–2000 pg/mL. Serum resistin levels were analyzed using a rat resistin ELISA kit (Cat. No.: FY‐ER2599, Lot No. KDWD7751203517), with a measurement range of 0.32–20 ng/mL. Serum visfatin concentrations were assessed using the rat visfatin ELISA kit (Cat. No.: FY‐ER3215, Lot No. KDWOI951203518) and the method's measurement range was determined to be 0.16–10 ng/mL. Serum insulin levels were measured using a rat insulin ELISA kit (Cat. No.: FY‐ER5259, Lot No. KDD8GL51203521) and the method's measurement range was 6.25–400 pg/mL. However, since the insulin values obtained in the groups were found to be above this range, the samples were diluted 1:3 with the kit buffer solution and reanalyzed. The obtained results were multiplied by the dilution factor to calculate the actual concentration values.

### 2.5 Anthropometric Measurements

#### 2.5.1 Examination of Abdominal Circumference, Chest Circumference, and Body Lengths

The body length of the rats was measured from head (nose region) to anus using a tape measure (Novelli et al. 2007). Abdominal circumference was measured by placing a measuring tape just in front of the forelimbs, while chest circumference was measured just behind the forelimbs (Mehmedagic et al. 2025). Measurements were performed using a flexible centimeter tape.

#### 2.5.2 Examination of Body Weight and BMI

The body weight of the rats was measured at the same time at the beginning of the experiment and once a week during the experiment using an electronic scale with decimal accuracy (ACCULAB VI‐I 200) and the change in body weight was calculated and recorded. Body weight gains were evaluated weekly. The BMI of the rats was calculated according to the following formula and those with a BMI of 0.45–0.68 g/cm^2^ were determined as normal and those with a BMI > 0.68 g/cm^2^ were considered obese (Novelli et al. 2007).

$$\mathrm{BMI}\;\left(g/{\mathrm{cm}}^{2}\right)=\text{body weight}/\text{Naso}-\text{anal}\;{\text{length}}^{2}$$

### 2.6 Analysis of Feed Consumption

Throughout the study period, the rats' feed intake was recorded daily using a precision balance sensitive to 0.1 g. The total amount of feed consumed by the animals was calculated by subtracting the remaining amount from the given amount on a daily basis, ad libitum. Feed intake was recorded at the cage level (two cages per group, five rats per cage) and therefore represents a cage level rather than an individual level variable.

### 2.7 Statistical Analysis

The sample size was determined in accordance with the 3Rs principles (Replacement, Reduction and Refinement) and an a priori power analysis performed using G*Power. Based on an effect size of 0.50, a significance level (*α*) of 0.05 and a statistical power of 80%, the required total sample size was calculated as 63 animals for seven groups (*n* = 9 per group). To account for potential animal loss, the sample size was increased by approximately 10%, resulting in 10 animals per group. Statistical analysis was conducted using The IBM Statistical Package for the Social Sciences (SPSS) version 26. Normality was assessed using the Shapiro–Wilk and Kolmogorov–Smirnov tests, as well as skewness and kurtosis values. Skewness and kurtosis values between −2 and +2 were considered sufficient for a normal distribution (George and Mallery 2024). To examine differences between groups, a one‐way analysis of variance (ANOVA) was performed, followed by Tukey's post hoc multiple comparison test. Results are presented as mean ± SEM. Feed intake was recorded and analyzed at the cage level (*n* = 2 cages per group) and is therefore presented as mean ± SD. When the groups were compared, the significance levels were determined as *p* < 0.05, *p* < 0.01 and *p* < 0.001, respectively. Graphs were created using GraphPad Prism 8.00 software.

## 3 Results

### 3.1 Biochemical Results

#### 3.1.1 Changes in Adipokines and Insulin Levels

**FIGURE 1:**
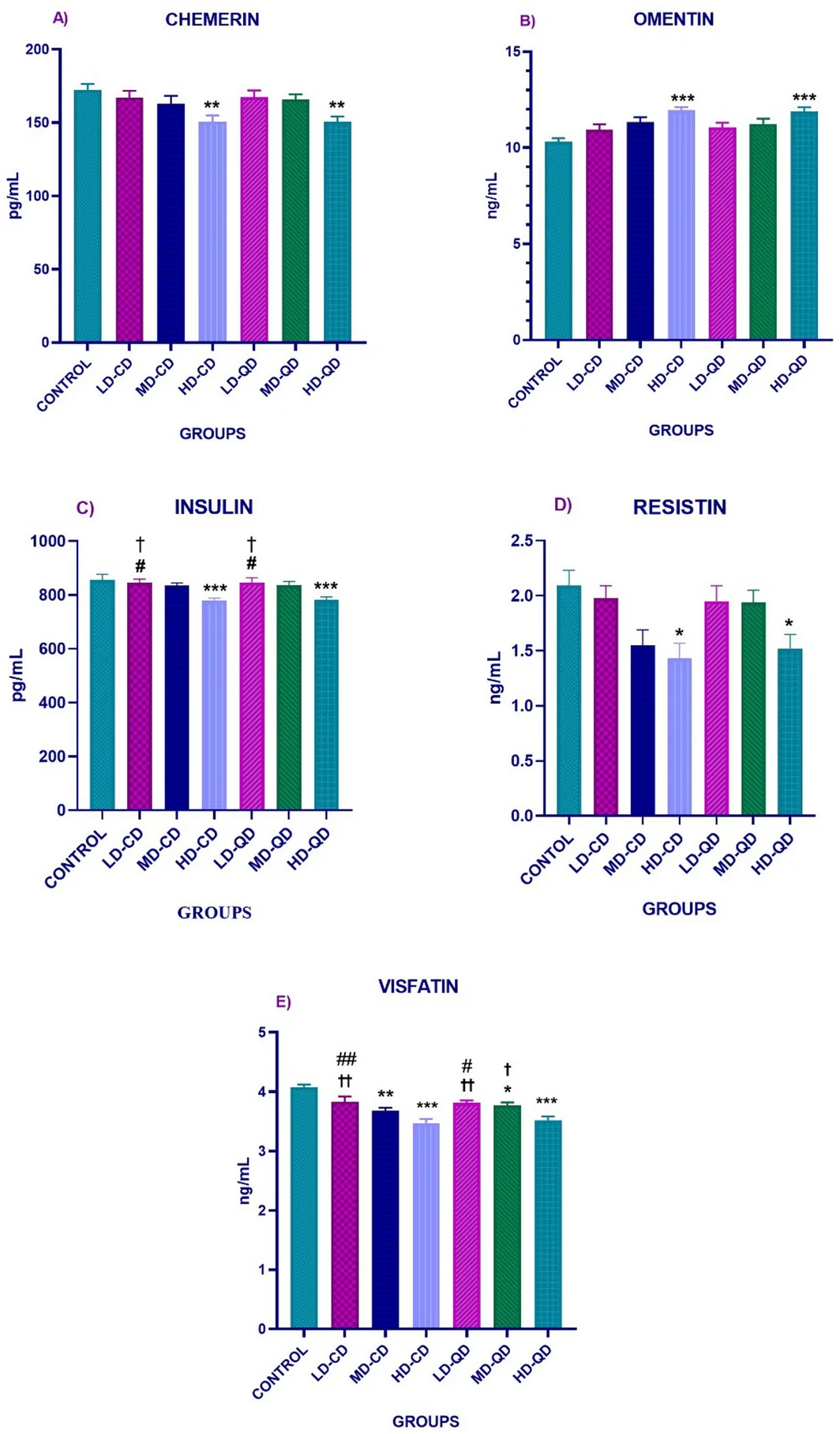
Chemerin levels (A), omentin levels (B), insulin levels (C), resistin levels (D), visfatin levels (E) of serum. HD‐CD, high‐dose chia diet; HD‐QD, high‐dose quinoa diet; LD‐CD, low‐dose chia diet; LD‐QD, low‐dose quinoa diet; MD‐CD, moderate‐dose chia diet; MD‐QD, moderate‐dose quinoa diet. *p < 0.05, **p < 0.01, ***p < 0.001 versus control group. ^#^ *p* < 0.05, ^##^ *p* < 0.01 versus HD‐QD, ^†^ *p* < 0.05, ^††^ *p* < 0.01 versus HD‐CD. Statistical comparisons were made using one‐way ANOVA followed by Tukey's test. The values represent as mean ± SEM.

Serum adipokine and insulin levels differed significantly among the groups (Figure 1). Compared with the control group, 20% chia and 20% quinoa supplementation significantly reduced serum chemerin (both *p* < 0.01), resistin (both *p* < 0.05) and insulin (both *p* < 0.001) and significantly increased serum omentin (both *p* < 0.001), whereas 5% and 10% supplementation had no significant effect on these parameters. Serum visfatin was significantly reduced by 10% and 20% chia (*p* < 0.01 and *p* < 0.001) and by 10% and 20% quinoa (*p* < 0.05 and *p* < 0.001), but not by 5% supplementation. In addition, relative to the 5% groups, the 20% chia and 20% quinoa groups showed significantly lower visfatin (*p* < 0.05) and insulin (*p* < 0.05), and 20% chia also showed lower visfatin than 10% quinoa (*p* < 0.05).

### 3.2 Feed Consumption Results

Table 3 shows the average daily feed intake of rats during the 6‐week follow‐up period in this study. Throughout the intervention, the HD‐CD and HD‐QD groups showed the lowest daily feed intake, whereas the control and LD‐QD groups showed the highest. It was found that the amount of feed consumed by the HD‐CD and HD‐QD groups was lower than that of the control group in the third, fourth, fifth, and sixth weeks (all *p* < 0.05). It was found that the amount of feed consumed by the HD‐CD and HD‐QD groups was lower compared to the LD‐QD group in the sixth week (both *p* < 0.05). In the second week, the overall ANOVA was significant, but the post hoc test did not identify any significant pairwise difference between the groups.

**TABLE 3:** Feed consumption of rats by week (g/day).

Weeks	Groups							*p*
	Control	LD‐CD	MD‐CD	HD‐CD	LD‐QD	MD‐QD	HD‐QD	Groups
1	24.39 ± 2.38	21.89 ± 3.48	24.47 ± 0.89	19.75 ± 1.27	23.39 ± 1.69	21.28 ± 0.19	20.21 ± 1.18	0.155
2	29.12 ± 1.52	24.81 ± 3.03	26.09 ± 0.60	22.85 ± 0.82	28.24 ± 1.13	24.45 ± 2.11	23.65 ± 0.15	0.041
3	29.44 ± 0.17^b^	25.54 ± 0.57^ab^	27.67 ± 0.74^ab^	23.79 ± 0.12^a^	28.96 ± 0.48^b^	24.73 ± 2.52^ab^	23.88 ± 1.64^a^	0.008
4	29.99 ± 0.89^b^	26.64 ± 1.94^ab^	27.98 ± 0.31^ab^	24.53 ± 1.97^a^	28.95 ± 0.12^ab^	25.96 ± 1.16^ab^	24.25 ± 1.54^a^	0.022
5	30.57 ± 0.42^b^	27.26 ± 2.57^ab^	28.05 ± 0.44^ab^	25.02 ± 1.01^a^	29.16 ± 0.55^ab^	26.69 ± 0.35^ab^	25.19 ± 1.41^a^	0.020
6	30.29 ± 0.19^b^	27.84 ± 1.92^ab^	28.76 ± 0.62^ab^	25.55 ± 0.65^a^	30.47 ± 0.35^b^	26.65 ± 0.56^ab^	25.92 ± 1.54^a^	0.009

*Note:* Values are presented as mean ± SD of two cages per group (*n* = 2 cages, 5 rats per cage). Data were statistically analyzed using one‐way ANOVA and Tukey post hoc test, where significant differences were labeled with distinct letter (a, b).

Abbreviations: HD‐CD, high‐dose chia diet; HD‐QD, high‐dose quinoa diet; LD‐CD, low‐dose chia diet; LD‐QD, low‐dose quinoa diet; MD‐CD, moderate‐dose chia diet; MD‐QD, moderate‐dose quinoa diet.

### 3.3 Anthropometric Measurement Results

#### 3.3.1 Body Weight Measurement Results

Table 4 shows the weekly body weights of the rats in the study groups. There was no significant difference in baseline body weight between the study groups. No significant difference in body weight was observed between the groups in the first and second weeks. After 6 weeks of dietary interventions, the groups showed significant increases compared to their baseline body weights.

**TABLE 4:** Body weight of rats by week (g).

Weeks	Groups							*p*
	Control	LD‐CD	MD‐CD	HD‐CD	LD‐QD	MD‐QD	HD‐QD	Groups
0	220.80 ± 6.63	220.60 ± 5.36	219.60 ± 5.09	221.00 ± 4.10	224.00 ± 4.25	221.60 ± 3.91	219.60 ± 4.47	0.997
1	243.40 ± 9.06	256.00 ± 5.30	245.60 ± 7.21	234.20 ± 6.25	249.56 ± 3.46	246.00 ± 6.07	238.80 ± 5.18	0.295
2	279.20 ± 9.83	277.60 ± 6.99	274.60 ± 7.59	254.00 ± 7.13	280.00 ± 2.28	271.20 ± 6.13	265.20 ± 5.67	0.112
3	314.70 ± 11.59^c^	291.00 ± 5.94^bc^	284.60 ± 8.20^abc^	257.60 ± 6.36^a^	293.33 ± 2.40^bc^	276.80 ± 7.32^ab^	271.00 ± 6.70^ab^	0.000
4	337.70 ± 9.82^c^	306.20 ± 6.62^bc^	300.00 ± 8.73^ab^	269.20 ± 6.45^a^	308.89 ± 3.07^bc^	295.20 ± 8.98^ab^	284.80 ± 6.99^ab^	0.000
5	342.10 ± 12.45^c^	331.10 ± 6.70^bc^	304.20 ± 8.60^ab^	289.20 ± 7.35^a^	328.11 ± 3.85^bc^	314.40 ± 11.01^abc^	303.70 ± 6.74^ab^	0.001
6	358.50 ± 10.98^c^	341.70 ± 6.95^bc^	319.50 ± 8.49^ab^	301.20 ± 7.69^a^	340.22 ± 4.25^bc^	330.10 ± 11.60^abc^	318.60 ± 6.90^ab^	0.000

*Note:* Values are presented as mean ± SEM (*n* = 10). Data were statistically analyzed using one‐way ANOVA and Tukey post hoc test, where significant differences were labeled with distinct letter (a, b, c).

Abbreviations: HD‐CD, high‐dose chia diet; HD‐QD, high‐dose quinoa diet; LD‐CD, low‐dose chia diet; LD‐QD, low‐dose quinoa diet; MD‐CD, moderate‐dose chia diet; MD‐QD, moderate‐dose quinoa diet.

Statistically significant differences in body weight levels were found between the groups in the third, fourth, fifth, and sixth weeks. In the third week, the mean body weights of the control, LD‐CD, and LD‐QD groups were higher compared to the HD‐CD group (*p* < 0.001, *p* < 0.05, *p* < 0.05, respectively). The body weights of the MD‐QD and HD‐QD groups were significantly lower compared to the control group (*p* < 0.05, *p* < 0.01, respectively). In the fourth week, the mean body weights of the control, LD‐CD, and LD‐QD groups were higher compared to the HD‐CD group (*p* < 0.001, *p* < 0.05, *p* < 0.01). The mean body weights of the MD‐CD, MD‐QD, and HD‐QD groups were significantly lower compared to the control group. At week five, the mean body weights of the control, LD‐CD, and LD‐QD groups were higher compared to the HD‐CD group (*p* < 0.01, *p* < 0.05, *p* < 0.05, respectively). The mean body weights of the MD‐CD and HD‐QD groups were significantly lower compared to the control group (both *p* < 0.05). At week six, the mean body weights of the control, LD‐CD, and LD‐QD groups were higher compared to the HD‐CD group (*p* < 0.001, *p* < 0.05, *p* < 0.05, respectively). The mean body weights of the MD‐CD and HD‐QD groups were significantly lower compared to the control group (both *p* < 0.05).

**FIGURE 2:**
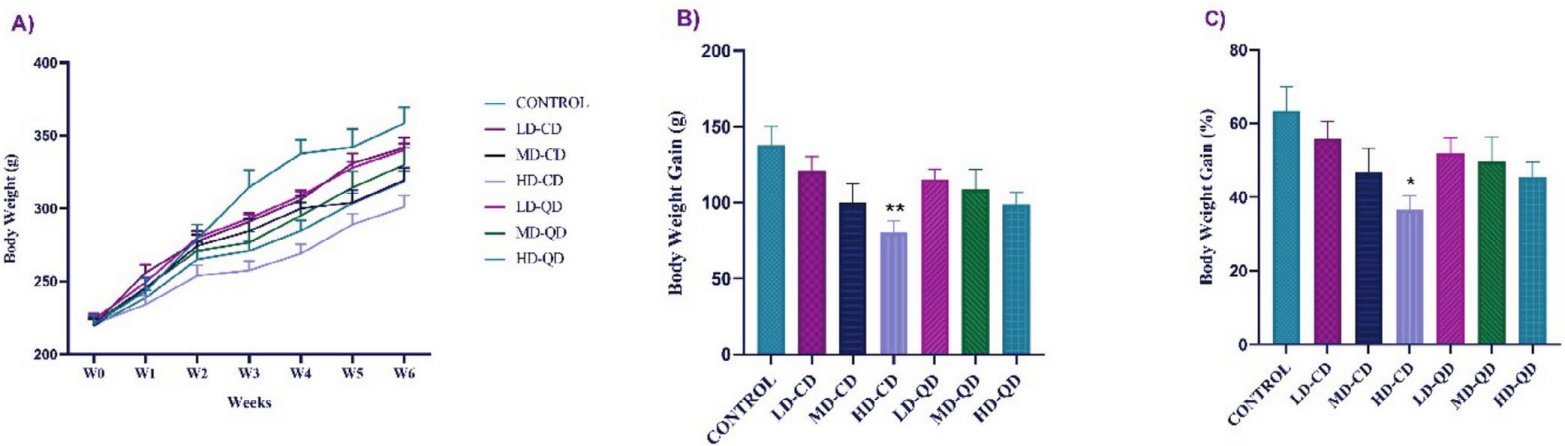
(A) Body weight, (B) Body weight gain (g), (C) Body weight gain (%). HD‐CD, high‐dose chia diet; HD‐QD, high‐dose quinoa diet; LD‐CD, low‐dose chia diet; LD‐QD, low‐dose quinoa diet; MD‐CD, moderate‐dose chia diet; MD‐QD, moderate‐dose quinoa diet. Control versus all groups *p < 0.05, **p < 0.01 versus control group. Statistical comparisons were made using one‐way ANOVA followed by Tukey's test. The values represent as mean ± SEM (*n* = 10).

The weekly body weights (g) of the rats in the study groups are shown in Figure 2A, body weight gain (g) in Figure 2B and body weight gain (%) in Figure 2C. ANOVA analysis of the body weight gain (g) levels revealed statistically significant differences between the groups. The body weight gain (g) of the HD‐CD group was significantly lower compared to the control group (*p* < 0.01). The body weight gain (%) of the HD‐CD group was significantly lower compared to the control group (*p* < 0.05).

#### 3.3.2 BMI Measurement Results

Table 5 shows the weekly BMI values of rats in the study groups. Initially, there was no significant difference in BMI between the study groups (*p* > 0.05). At the end of the first week, the BMI values of the MD‐CD, MD‐QD and HD‐QD groups were significantly lower than the control group (*p* < 0.05, *p* < 0.01, *p* < 0.001, respectively). At the end of the second week, the BMI values of the LD‐CD, MD‐CD, HD‐CD, LD‐QD, MD‐QD, and HD‐QD groups were significantly lower than the control group (*p* < 0.01, *p* < 0.01, *p* < 0.001, *p* < 0.001, *p* < 0.01, *p* < 0.001, respectively). At the end of the third week, the BMI values of the LD‐CD, MD‐CD, HD‐CD, LD‐QD, MD‐QD, and HD‐QD groups were significantly lower than the control group (*p* < 0.05, *p* < 0.001, *p* < 0.001, *p* < 0.01, *p* < 0.01, *p* < 0.001, respectively). In the fourth, fifth and sixth weeks, BMI values showed statistically significant differences between the groups (*p* < 0.001). The BMI values of the LD‐CD, MD‐CD, HD‐CD, LD‐QD, MD‐QD, and HD‐QD groups were significantly lower than the control group (*p* < 0.001 for all groups).

**TABLE 5:** BMI of rats by week (g/cm^2^).

Weeks	Groups							*p*
	Control	LD‐CD	MD‐CD	HD‐CD	LD‐QD	MD‐QD	HD‐QD	Groups
0	0.64 ± 0.02	0.63 ± 0.01	0.65 ± 0.01	0.62 ± 0.01	0.63 ± 0.01	0.64 ± 0.01	0.65 ± 0.01	0.265
1	0.66 ± 0.02^b^	0.60 ± 0.02^ab^	0.59 ± 0.01^ab^	0.59 ± 0.01^ab^	0.59 ± 0.02^ab^	0.57 ± 0.01^a^	0.55 ± 0.01^a^	0.001
2	0.66 ± 0.02^b^	0.58 ± 0.01^a^	0.59 ± 0.01^a^	0.55 ± 0.01^a^	0.57 ± 0.02^a^	0.58 ± 0.02^a^	0.56 ± 0.01^a^	0.000
3	0.67 ± 0.02^b^	0.60 ± 0.01^a^	0.57 ± 0.01^a^	0.55 ± 0.01^a^	0.58 ± 0.01^a^	0.58 ± 0.02^a^	0.57 ± 0.01^a^	0.000
4	0.67 ± 0.01^b^	0.56 ± 0.01^a^	0.53 ± 0.01^a^	0.52 ± 0.01^a^	0.55 ± 0.01^a^	0.54 ± 0.01^a^	0.53 ± 0.01^a^	0.000
5	0.65 ± 0.02^b^	0.57 ± 0.01^a^	0.52 ± 0.01^a^	0.54 ± 0.01^a^	0.57 ± 0.01^a^	0.56 ± 0.01^a^	0.56 ± 0.01^a^	0.000
6	0.65 ± 0.01^b^	0.56 ± 0.01^a^	0.53 ± 0.01^a^	0.54 ± 0.01^a^	0.56 ± 0.01^a^	0.56 ± 0.02^a^	0.54 ± 0.01^a^	0.000

*Note:* Values are presented as mean ± SEM (*n* = 10). Data were statistically analyzed using one‐way ANOVA and Tukey post hoc test, where significant differences were labeled with distinct letter (a, b).

Abbreviations: HD‐CD, high‐dose chia diet; HD‐QD, high‐dose quinoa diet; LD‐CD, low‐dose chia diet; LD‐QD, low‐dose quinoa diet; MD‐CD, moderate‐dose chia diet; MD‐QD, moderate‐dose quinoa diet.

#### 3.3.3 Abdominal Circumference Measurement Results

Abdominal circumference measurements of rats in the study groups according to weeks are shown in Table 6. At baseline, no significant difference was observed in abdominal circumference measurements between the study groups in the first, second, third, fourth, and fifth weeks. Following the 6 week dietary intervention, the groups showed significant increases in abdominal circumference compared with their baseline values. At the sixth week, the abdominal circumference of the HD‐CD group was significantly lower compared to the control group (*p* < 0.05). The abdominal circumference of the HD‐CD and MD‐QD groups was significantly lower compared to the LD‐CD group (*p* < 0.001, *p* < 0.05).

**TABLE 6:** Abdominal circumference of rats by week (cm).

Weeks	Groups							*p*
	Control	LD‐CD	MD‐CD	HD‐CD	LD‐QD	MD‐QD	HD‐QD	Groups
0	15.10 ± 0.26	15.05 ± 0.25	15.25 ± 0.29	14.70 ± 0.21	15.00 ± 0.22	15.40 ± 0.47	15.10 ± 0.28	0.772
1	17.10 ± 0.23	16.75 ± 0.27	16.85 ± 0.22	16.40 ± 0.27	17.00 ± 0.14	16.40 ± 0.18	17.15 ± 0.15	0.072
2	16.50 ± 0.28	16.65 ± 0.31	16.40 ± 0.29	16.15 ± 0.43	16.22 ± 0.17	16.45 ± 0.37	16.10 ± 0.19	0.853
3	16.65 ± 0.22	16.50 ± 0.34	16.55 ± 0.31	16.20 ± 0.31	16.44 ± 0.17	15.85 ± 0.27	15.95 ± 0.19	0.249
4	16.40 ± 0.12	16.20 ± 0.30	15.95 ± 0.28	15.55 ± 0.20	16.39 ± 0.11	15.70 ± 0.33	16.35 ± 0.26	0.082
5	17.35 ± 0.20	16.95 ± 0.30	17.30 ± 0.21	16.65 ± 0.20	16.72 ± 0.12	16.75 ± 0.31	16.70 ± 0.17	0.140
6	16.55 ± 0.23^bc^	17.05 ± 0.31^c^	16.45 ± 0.29^abc^	15.40 ± 0.23^a^	16.44 ± 0.15^abc^	15.75 ± 0.35^ab^	16.35 ± 0.22^abc^	0.001

*Note:* Values are presented as mean ± SEM (*n* = 10). Data were statistically analyzed using one‐way ANOVA and Tukey post hoc test, where significant differences were labeled with distinct letter (a, b, c).

Abbreviations: HD‐CD, high‐dose chia diet; HD‐QD, high‐dose quinoa diet; LD‐CD, low‐dose chia diet; LD‐QD, low‐dose quinoa diet; MD‐CD, moderate‐dose chia diet; MD‐QD, moderate‐dose quinoa diet.

#### 3.3.4 Chest Circumference Measurement Results

Chest circumference measurements of rats in the study groups according to weeks are shown in Table 7. A significant difference was found in chest circumference measurements between the study groups in the first and fourth weeks. After 6 weeks of dietary intervention, the groups showed significant increases in chest circumference measurements compared to their baseline chest circumference measurements. In the first week, the chest circumference of the LD‐CD group was significantly lower than that of the MD‐QD group (*p* < 0.05). The chest circumference of the HD‐CD group was significantly lower than that of the MD‐QD group (*p* < 0.01). In the fourth week, the chest circumference of the HD‐CD group was significantly lower than that of the control group (*p* < 0.05).

**TABLE 7:** Chest circumference of rats by week (cm).

Weeks	Groups							*p*
	Control	LD‐CD	MD‐CD	HD‐CD	LD‐QD	MD‐QD	HD‐QD	Groups
0	13.65 ± 0.21	13.80 ± 0.30	13.75 ± 0.32	13.40 ± 0.21	13.56 ± 0.21	13.60 ± 0.19	13.25 ± 0.23	0.701
1	13.90 ± 0.24^ab^	13.55 ± 0.24^a^	14.15 ± 0.20^ab^	13.45 ± 0.17^a^	14.28 ± 0.26^ab^	14.65 ± 0.32^b^	14.45 ± 0.17^ab^	0.004
2	14.45 ± 0.19	14.05 ± 0.20	14.20 ± 0.13	14.15 ± 0.18	14.11 ± 0.14	14.40 ± 0.23	14.25 ± 0.13	0.659
3	15.10 ± 0.14	14.30 ± 0.28	14.75 ± 0.21	14.50 ± 0.23	14.78 ± 0.17	14.75 ± 0.25	14.80 ± 0.13	0.217
4	15.00 ± 0.15^b^	14.40 ± 0.24^ab^	14.25 ± 0.11^ab^	14.10 ± 0.14^a^	15.00 ± 0.14^b^	14.55 ± 0.35^ab^	14.65 ± 0.18^ab^	0.018
5	15.65 ± 0.15	15.25 ± 0.33	15.60 ± 0.12	15.15 ± 0.29	14.83 ± 0.20	15.55 ± 0.26	14.90 ± 0.16	0.070
6	15.70 ± 0.23	15.10 ± 0.21	15.50 ± 0.21	14.95 ± 0.28	15.50 ± 0.08	15.40 ± 0.19	15.50 ± 0.10	0.133

*Note:* Values are presented as mean ± SEM (*n* = 10). Data were statistically analyzed using One‐way ANOVA and Tukey post hoc test, where significant differences were labeled with distinct letter (a,b).

Abbreviations: HD‐CD, high‐dose chia diet; HD‐QD, high‐dose quinoa diet; LD‐CD, low‐dose chia diet; LD‐QD, low‐dose quinoa diet; MD‐CD, moderate‐dose chia diet; MD‐QD, moderate‐dose quinoa diet.

## 4 Discussion

The increasing consumption in recent years of diets high in calories but low in nutritional value has become a major nutritional concern, as it may contribute to an increased risk of chronic diseases such as hidden hunger, obesity, diabetes, and cardiovascular disease (Ministry of Health of Türkiye 2022). Chia ( *Salvia hispanica* L.) and quinoa ( *Chenopodium quinoa* Willd.) seeds are two important pseudocereals that stand out due to their high dietary fiber content, unsaturated fatty acids, and bioactive nutrient compounds associated with beneficial metabolic effects (Balakrishnan et al. 2025; Xi et al. 2024). Although the potential health effects of chia and quinoa seeds have been extensively studied, evidence regarding their effects on adipokine regulation and related metabolic biomarkers (anthropometric measurements) remains limited.

The lower feed intake observed in the high inclusion groups may reflect differences in the overall diet formulations, including their fiber and fat contents as well as possible properties of chia and quinoa related to satiety. Chia seeds demonstrate their potential to increase satiety through their high dietary fiber content and gel‐forming properties. These properties can increase the viscosity of digestive fluids, delay gastric emptying and prolong the feeling of fullness (Wolever et al. 2023). Quinoa, on the other hand, may exhibit potential satiety enhancing effects by stimulating postprandial cholecystokinin release through its content of saponins, dietary fiber and phenolic compounds (Mithila and Khanum 2015; Simnadis et al. 2015). Studies have shown that 7 and 14 g of chia seeds create a short term feeling of fullness and reduce the amount of food consumed (Ayaz et al. 2017). It was found that adding quinoa to rat diets led to a significant reduction in average daily food intake (Mithila and Khanum 2015).

The gradual increase in body weight observed across all groups over a 6 week period is consistent with the normal growth pattern during the rats' developmental stages (Cossio‐Bolanos et al. 2013). Rats consuming a diet supplemented with a high dose of chia seeds were found to have lower body weights. This suggests that the potential effects of chia seeds on body weight may become more pronounced as dietary intake increases. However, the lower feed intake observed in this group may also have contributed to the effect, alongside the potential influence of chia. This observation may be related to the high dietary fiber content of chia seeds. The high dietary fiber content of chia seeds may have potential effects that increase satiety, reduce calorie intake, and consequently limit body weight gain (Grancieri et al. 2019). It has also been suggested that chia, due to its lower calorie content and rich omega‐3 fatty acid content, contributes to body weight regulation by limiting lipid accumulation and promoting fat oxidation (Muhsin et al. 2024; Pandurangan et al. 2020). Previous experimental studies have shown that these findings are consistent across chia doses ranging from 5% to 15% and intervention periods of five to 8 weeks and that they also occur in healthy, nonobese animals (da Silva et al. 2016; Fernandez‐Martinez et al. 2019; Rashid et al. 2026; Sebo 2017).

The lower BMI values observed with chia and quinoa supplementation at different doses may be associated with the bioactive components of these seeds; however, differences in the overall composition of the experimental diets and feed intake may also have contributed to these changes. It has been reported that quinoa exerts its anti‐obesity effects primarily through the phytoecdysteroid 20‐hydroxyecdysone (20‐HE) in its composition and that 20‐HE exerts this effect by interacting with vitamin D receptors and regulating the expression of genes involved in lipid accumulation (Foucault et al. 2012). Quinoa also contains dietary fiber, saponins and phenolic compounds. These components exhibit potential effects that increase satiety and reduce food intake through mechanisms related to appetite regulation (Simnadis et al. 2015). Similarly, chia seeds may increase satiety by promoting gel formation in the gastrointestinal tract due to their high dietary fiber content; furthermore, it has been demonstrated that in combination with an appropriate omega‐3 fatty acid profile, they suppress appetite, limit fat accumulation and increase energy expenditure (Jeelani et al. 2023). These proposed mechanisms are supported by previous clinical and experimental studies reporting a reduction in BMI following chia and quinoa supplementation (de Cássia Lovato et al. 2022; Doğan Güney and Göbel 2025; Fateh et al. 2024). Although many clinical and experimental studies have reported a decrease in BMI following quinoa supplementation (Kothari and Jeyaraj 2016; Melios et al. 2025; Noratto et al. 2019; Pourshahidi et al. 2018, 2020; Abellán Ruiz et al. 2017), there are also studies that have failed to find a significant effect (Atefi et al. 2024; Karimian et al. 2021; Navarro‐Perez et al. 2017). It is thought that the conflicting results may be related to differences in the form of quinoa used, the supplement dose, the duration of the intervention and the metabolic characteristics of the study population. However, since this study was conducted on healthy, growing rats, it is thought that lower feed intake may have contributed to the lower body weight gain and reduced BMI observed in the high‐dose groups.

During the intervention period, abdominal circumference and chest circumference increased; this is consistent with the normal growth pattern of rats during development (Cossio‐Bolanos et al. 2013). The smaller increases in anthropometric measurements observed in the high‐dose chia group may suggest a modest influence of high‐dose chia supplementation on body weight‐related outcomes. Nevertheless, the lower feed intake in this group may also have contributed to these findings and given that body composition was not directly assessed, this interpretation should be approached with caution. The potential positive effects of chia seeds on anthropometric measurements may be related to their high content of dietary fiber, omega‐3 fatty acids, and phenolic compounds, which could contribute to improved anthropometric outcomes by increasing satiety and reducing energy intake (Jeelani et al. 2023). In line with these findings, clinical studies have reported improvements in anthropometric parameters, particularly waist circumference, following chia supplementation at doses of 30, 35, and over 35 g under various intervention conditions (Saadh et al. 2025; Shahparvari and Nasrollahzadeh 2024). In our study, however, neither abdominal nor chest circumference was significantly affected following quinoa supplementation. It is thought that this finding may be related to the use of healthy, growing rats, in which anthropometric responses to dietary interventions are generally less pronounced than those reported in cases of obesity or other metabolic disorders (Diaz‐Rizzolo et al. 2022; Huang et al. 2025; Karimian et al. 2021). Similarly, randomized controlled trials conducted in metabolically stable individuals have shown that there was no significant change in anthropometric measurements following quinoa supplementation (Mohsin et al. 2023; Navarro‐Perez et al. 2017). These findings suggest that any anthropometric changes resulting from quinoa supplementation may depend on factors such as the study population, supplementation dose, duration of intervention, and the form of quinoa used.

Omentin‐1, secreted by visceral adipose tissue, is an important adipokine that plays a role in metabolic regulation and exhibits anti‐inflammatory properties. It has been demonstrated that higher circulating levels of omentin‐1 are associated with favorable metabolic traits and support its potential role as a biomarker of metabolic status (Karvane et al. 2024). Increased serum omentin concentrations associated with high‐dose chia and quinoa supplementation may reflect a favorable modulation of the circulating adipokine profile. It is thought that the potential effects of these seeds on serum omentin concentrations may be attributed to their bioactive nutrient content. Studies have shown that omega‐3 fatty acids may positively influence adipokine regulation and are associated with higher circulating concentrations of omentin (Fauzi et al. 2025; Hussein et al. 2024; Siriwardhana et al. 2013). Similarly, it has been shown that the anti‐inflammatory effects of dietary polyphenols and bioactive compounds may contribute to adipokine homeostasis (Siriwardhana et al. 2013). These findings suggest that the bioactive compounds in chia and quinoa seeds may have contributed to the increased serum omentin‐1 concentrations observed in the present study.

Resistin is an adipokine secreted by adipose tissue and positively associated with insulin resistance, chronic inflammation, and various metabolic disorders. Studies have suggested that reducing circulating resistin concentrations is a potential strategy for alleviating inflammatory and metabolic disorders (Deb et al. 2021; Tripathi et al. 2020). In the current study, the decrease in serum resistin concentrations observed with high‐dose chia and quinoa supplementation may reflect a positive modulation of the circulating adipokine profile. The potential effects of chia and quinoa on serum resistin concentrations may be related to the bioactive composition of these grains. Previous studies have associated dietary polyphenol‐rich plant extracts as well as olive oil and propolis, which are high in phenolic compounds, with reduced circulating resistin concentrations, and it has been reported that these substances may positively influence resistin levels (Boix‐Castejon et al. 2018; Graf et al. 2013; Machowetz et al. 2008; Malek Mahdavi et al. 2026). Clinical studies have also demonstrated a significant negative correlation between omega‐3 fatty acids and circulating resistin levels (Reyes‐Barrera et al. 2022). Overall, these findings suggest that the polyphenols and omega‐3 fatty acids found in chia and quinoa may have contributed to lower serum resistin concentrations.

Elevated circulating concentrations of chemerin, which plays a role in adipogenesis, glucose homeostasis, and adipose tissue function, have been associated with obesity, inflammation, and metabolic disorders. Low levels of circulating chemerin are generally associated with reduced inflammation and metabolic disorders (Helfer and Wu 2018; Ozyalcin and Sanlier 2025; Su et al. 2021). Our study hypothesizes that the lower serum chemerin concentrations observed following high‐dose chia and quinoa supplementation may be related to the anti‐inflammatory and antioxidant properties of these seeds. Previous studies have shown a positive correlation between the inflammatory potential of the diet and circulating chemerin concentrations and that pro‐inflammatory dietary models are associated with higher chemerin levels (Mirmajidi et al. 2019; Suhett et al. 2021). However, while it has been reported that polyphenols, omega‐3 fatty acids, alpha‐lipoic acid (which has high antioxidant capacity), and other antioxidant components lower chemerin levels (El‐Shawwa 2019; Mirmajidi et al. 2019; Prieto‐Hontoria et al. 2016; Yu et al. 2012), high consumption of red meat and sugary beverages, which are associated with high inflammatory markers, and low consumption of dairy products have been linked to increased chemerin levels (Koelman et al. 2021). When the available evidence is evaluated as a whole, it is thought that the antioxidant and anti‐inflammatory components of chia and quinoa may have contributed to lower serum chemerin concentrations.

Visfatin is an adipokine that is primarily expressed in visceral adipose tissue and plays potential roles in metabolic and inflammatory processes. Given that circulating visfatin concentrations are associated with obesity and metabolic disorders, changes in visfatin levels in the circulation are thought to provide useful information about metabolic status (De Luis et al. 2011; Jain and Goel 2025). In the present study, the lower serum visfatin concentrations observed following chia and quinoa supplementation may be related to the high polyphenol and unsaturated fatty acid content of these pseudocereals. Studies have reported that visfatin concentrations are influenced by dietary factors; it has been suggested that these factors may reduce circulating visfatin concentrations by suppressing visfatin expression in adipose tissue or by increasing visfatin sensitivity (Saboori et al. 2015). Similarly, it has been reported that polyphenol‐rich foods including extra virgin olive oil, resveratrol, and fruits rich in phytochemicals reduce serum visfatin concentrations and, in some cases, visfatin gene expression (Asadi et al. 2015; Erkılıç and Bayraktar 2025; Santangelo et al. 2018). Furthermore, it has been shown that diets rich in unsaturated fatty acids are also associated with lower circulating visfatin concentrations (Haghighatdoost et al. 2012). These findings suggest that the polyphenols and unsaturated fatty acids found in chia and quinoa may have contributed to the lower serum visfatin concentrations observed in our study.

When adipokines are considered together, the increase in the anti‐inflammatory adipokine omentin and the decrease in the pro‐inflammatory adipokines chemerin, resistin and visfatin suggest a shift in the circulating adipokine profile toward a more favorable direction; this is biologically significant given the well‐known associations of these adipokines with inflammation, insulin signaling, appetite regulation and cardiometabolic risk and the current findings can be correlated with their potential physiological significance.

Insulin is a key anabolic hormone that plays a central role in the regulation of glucose and energy metabolism (Petersen and Shulman 2018). Previous experimental and clinical studies have reported lower fasting insulin concentrations following supplementation with chia seed powder, chia seed extracts, or quinoa seeds incorporated into the diet (Jangra et al. 2025; Omran et al. 2023; Ozcaliskan Ilkay et al. 2023; Rashid et al. 2026). Similarly, lower fasting serum insulin concentrations were observed in the higher dose chia and quinoa groups in the present study. The favorable effects of chia and quinoa on fasting insulin concentrations may be associated with their dietary fiber, omega‐3 fatty acid, and polyphenol contents, all of which have been associated with insulin regulation in previous studies. Overall, the available evidence suggests that the bioactive constituents of chia and quinoa may have contributed to the lower fasting serum insulin concentrations observed in the present study. However, since this study was conducted on healthy, growing rats, it is thought that lower feed intake may have contributed to the lower serum fasting insulin levels observed in the chia and quinoa supplemented groups.

This study has several limitations that should be considered when interpreting the findings. First, the adipokine profile was limited to omentin, visfatin, resistin, and chemerin, whereas other metabolically relevant adipokines, including leptin and adiponectin, were not evaluated. Although fasting insulin concentrations were measured following an overnight fast, fasting glucose, lipid profile, and liver function markers were not assessed, limiting a more comprehensive evaluation of metabolic status. Histological examination of metabolically relevant tissues, including adipose tissue, liver, and pancreas, was not performed; therefore, the relationship between circulating biomarkers and tissue level alterations remains to be established. Furthermore, feed intake was assessed at the cage level, and no pair‐fed control group was included. Consequently, the lower body weight gain, BMI, fasting insulin, and adipokine concentrations observed in the higher supplementation groups may have been influenced, at least in part, by differences in feed and energy intake. Although the diets were formulated to be isocaloric and isonitrogenous, the addition of chia or quinoa necessitated changes in other dietary components. Therefore, the observed effects cannot be attributed solely to chia or quinoa supplementation. Future studies incorporating pair‐fed controls, comprehensive dietary characterization, histological evaluation, and molecular analyses are warranted to clarify the mechanisms underlying the observed effects.

## 5 Conclusions

In this 6 week study, rats fed diets containing different levels of chia or quinoa showed differences in serum adipokines, fasting insulin, BMI, and other anthropometric outcomes. Among the two pseudocereals, 20% chia supplementation produced somewhat more pronounced effects on body weight and anthropometric measurements, whereas both seeds showed comparable effects on serum adipokine levels. However, the experimental diets differed in proximate composition, and the high inclusion groups consumed less feed than the control group. In the absence of pair‐fed controls, the findings primarily reflect the overall effects of the complete dietary formulations under ad libitum feeding conditions, and the specific contributions of chia and quinoa should therefore be interpreted with caution. Further studies using pair‐fed controls and comprehensively characterized diets are required before conclusions specific to individual ingredients or dietary recommendations can be made. As the study was conducted in healthy rats over a 6 week period, these results should be regarded as preclinical, and controlled human trials are required before dietary recommendations can be made. Clinical studies are needed to clearly define and demonstrate the applicability and efficacy of these effects in human populations as well as to determine effective doses, and these findings should be confirmed in human clinical trials.

## Author Contributions

**Kübra İspir:** conceptualization, investigation, writing – original draft. **Sevinç Yücecan:** conceptualization, methodology, formal analysis, writing – review and editing. **Ilyas Bozkurt:** investigation. **Halit Imik:** formal analysis, methodology, investigation, writing – review and editing.

## Funding

This research was part of the PhD thesis of Kübra İspir. The present study was supported by the Atatürk University Scientific Research Projects Coordination (grant no. THD‐2025‐15647).

## Ethics Statement

This study was approved by the Atatürk University Experimental Animal Ethics Committee (Document No.: E‐36643897‐000‐2500066231; Decision number: 45; Acceptance date: 25 February 2025). All experimental methods and procedures were conducted in accordance with the relevant ethical standards for animal research.

## Consent

The authors have nothing to report.

## Conflicts of Interest

The authors declare no conflicts of interest.

## Data Availability

The data that support the findings of this study are available from the corresponding author upon reasonable request.
